# Early cerebral amyloid angiopathy-related pathology is associated with localized functional brain connectivity

**DOI:** 10.1177/13872877261469189

**Published:** 2026-07-23

**Authors:** Nadieh Drenth, Suzanne E. van Dijk, Anne Hafkemeijer, Serge A.R.B. Rombouts, Jeroen van der Grond, Sanneke van Rooden

**Affiliations:** 1 Department of Radiology, 4501 Leiden University Medical Center, Leiden, The Netherlands; 2 4496 Institute of Psychology, Leiden University, Leiden, The Netherlands; 3 Leiden Institute for Brain and Cognition, Leiden, The Netherlands

**Keywords:** Alzheimer's disease, cerebral amyloid angiopathy, functional magnetic resonance imaging, neurovascular coupling

## Abstract

**Background:**

The mechanisms of early brain damage in cerebral amyloid angiopathy (CAA), a highly prevalent comorbid condition in Alzheimer's disease, are not completely understood. While current CAA diagnosis relies on late-stage MRI markers, impaired neurovascular coupling (NVC) has emerged as a promising early marker.

**Objective:**

To investigate whether early vascular changes in CAA are associated with functional brain consequences, we examined the association between NVC and various functional connectivity metrics.

**Methods:**

We analyzed MRI data from 93 older adults (71 ± 9 years old). A subgroup meeting Boston criteria v2.0 for possible or probable CAA (*n* = 46) was analyzed separately to investigate associations in confirmed CAA-related pathology. NVC (time to peak, time to baseline, and BOLD amplitude) was assessed in the occipital cortex using a visual stimulation task. Functional connectivity was assessed at multiple scales using resting-state fMRI, including within ten standard networks, between individual brain regions (edgewise analysis), and across the whole brain (graph theory).

**Results:**

General linear models, adjusted for age, sex, and clinical diagnosis, showed no significant associations between NVC and global connectivity metrics (networks and graph theory). Edgewise analyses revealed limited significant localized functional connections for each NVC measure. Findings were consistent across the total sample and the CAA subgroup.

**Conclusions:**

Early CAA-related pathology is associated with localized rather than global functional connectivity. While large-scale connectivity remains preserved, edgewise analyses indicate subtle localized functional alterations. This suggests that functional connectivity disruption may originate as small-scale deficits that only progress into widespread, global impairment in advanced disease stages.

## Introduction

Cerebral amyloid angiopathy (CAA) is a severe and progressive cerebrovascular disorder that is characterized by the accumulation of amyloid beta peptide deposits in the small to medium-sized cerebral blood vessels and leptomeningeal arteries, resulting in intracerebral hemorrhages, loss of neurological function and cognitive decline.^1^ CAA is a common, but often asymptomatic finding in the elderly: autopsy studies indicate a prevalence of 40% in non-demented elderly^2^ that rises to 60% in (vascular) demented elderly and even up to 98% in elderly with Alzheimer's disease.^2,3^

In vivo detection of CAA-related brain damage is currently facilitated through neuroimaging markers that indicate the presence and location of late-stage brain lesions, such as cerebral (micro)bleeds.^4^ While these markers of advanced disease are well-established, emerging evidence now points to earlier, more subtle pathological changes, specifically impaired neurovascular coupling. This impairment was first described in the occipital cortex of non-demented sporadic CAA patients during visual stimulation^5^ and was later found to be already present in the presymptomatic phase of Dutch-type CAA.^6^ Importantly, a four-year follow-up study showed that neurovascular coupling worsens over time, even without a concurrent increase in hemorrhagic lesions, underscoring its potential as an early and sensitive marker for CAA.^7^

To determine how early vascular changes in CAA affect brain function, the present study uses functional connectivity analysis. This method is highly suited for studying the intricate organization of brain networks that underpin sensory and cognitive processes.^8–11^ Alterations in these networks are recognized as sensitive, early indicators of brain dysfunction in many neurodegenerative diseases,^12–15^ often preceding overt cognitive decline.^16,17^ Crucially, alterations in functional connectivity have already been identified in CAA, both in manifest sporadic and Dutch-type forms, and even emerging in the presymptomatic phase of the disease.^18,19^ Therefore, by examining the association between neurovascular coupling and various functional connectivity metrics, we aim to better understand the mechanisms of early CAA-related brain damage.

## Methods

### Participants and procedures

Participants were recruited from the memory clinics of the Leiden University Medical Center (Leiden, the Netherlands) and the Haaglanden Medical Center (The Hague, the Netherlands) after being referred to the hospital by their general practitioner or a medical specialist based on memory complaints. Patients were diagnosed at the memory clinics through a multidisciplinary consensus meeting using the National Institute of Neurological and Communicative Disorders and Stroke and Alzheimer's Disease and Related Disorders Association (NINCDS-ADRDA) criteria^20^ and eligible for this study if they were diagnosed with subjective cognitive impairment (SCI), mild cognitive impairment (MCI), probable Alzheimer's disease or mixed type dementia. Additionally, people without cognitive complaints between 40 and 90 years old were recruited via various advertisements.

All participants visited the LUMC to undergo an MRI scanning session. Neuropsychological assessment took place if this had not been done beforehand in the memory clinic. Participants recruited as cognitively normal were excluded if the neuropsychological assessment revealed any signs of cognitive impairment. For all participants, exclusion criteria were inability to give informed consent, any MRI contraindications or specific contraindications for performing a visual task in the MRI, being seizure within one year prior to scanning and non-correctable visual impairment. The Medical Ethical Committee of Leiden Den Haag Delft approved the study and written informed consent was obtained from all participants. The procedures followed were in accordance with institutional guidelines and the World Medical Association (WMA) Declaration of Helsinki.

### Image acquisition and processing

Participants were scanned at the Leiden University Medical Center on a 3-Tesla Philips Achieva MRI scanner (Philips Medical Systems, Best, the Netherlands) equipped with a standard 32-channel head coil. The scanning session included a high-resolution structural image, resting-state and task-based functional scans, and sequences for lesion detection.

A high-resolution three-dimensional T1-weighted (3D-T1) structural image was acquired with an echo time (TE) of 3.5 ms, a repetition time (TR) of 7.9 ms, a flip angle (FA) of 8°, a field of view (FOV) of 250 × 199 × 170 mm, and a voxel size of 1.1 × 1.1 × 1.1 mm, resulting in a scan duration of 4 min and 18 s. The imaging protocol also included lesion-detection scans, such as T2-weighted, T2*-weighted, and fluid-attenuated inversion recovery (FLAIR) images, which have been described elsewhere.^21^

Resting-state fMRI (RS-fMRI) scans were acquired with the following parameters: TR = 2200 ms, TE = 30 ms, FA = 80°, FOV = 220 × 220 × 114 mm, voxel size 2.75 × 2.75 × 2.75 mm, 38 slices, total scan duration of 7 min 29 s. During the resting-state scan, participants were instructed to keep their eyes closed and not to fall asleep. For the neurovascular coupling assessment, three visually stimulated fMRI scan series were acquired using a TR of 1500 ms, TE of 30 ms, FA of 75°, a 220 × 220 x 75 mm FOV, a voxel size of 2.75 × 2.75 × 2.75 mm, with 25 slices positioned over the occipital cortex for a total scan duration of 3 min 21 s. All fMRI acquisitions were preceded by two dummy scans for T1 stabilization purposes. During the visual stimulation task, a radial black and white checkerboard pattern flashed at 8 Hz for 20 s (stimulus) alternated with 28 s of gray screen (rest), as described previously.^5^ Three visually stimulated scan series were acquired in succession, each containing 4 stimulus-rest cycles. To ensure continuous attention, a central fixation dot randomly changed color, and participants were instructed to press a button with each change.

Following acquisition, all MR images were visually inspected for gross artifacts. The lesion detection sequences were then used to score MRI markers of CAA and general CSVD, including intracerebral hemorrhages, microbleeds, cortical superficial siderosis, dilated perivascular spaces in the centrum semiovale and basal ganglia, lacunar infarcts, and white matter hyperintensities as described previously.^21^ The detailed processing of the functional images is described in the following sections.

### Preprocessing of functional images

Preprocessing of fMRI images was performed using the fMRI Expert Analysis Tool (FEAT) as implemented in FMRIB's Software Library (FSL version 6.0).^22^ Functional images were skull-stripped using the brain extraction tool^23^ and motion corrected using MCFLIRT.^24^ Spatial smoothing was applied with an 8 mm Gaussian kernel for visually stimulated fMRIs and a 5 mm Gaussian kernel for resting-state fMRIs. High-pass temporal filtering at 100 s was applied. Resting-state fMRIs were additionally denoised with an independent component analysis (ICA)-based strategy for Automatic Removal of Motion Artifacts (ICA-AROMA).^25,26^ All functional images were spatially normalized to the 2 mm MNI152 standard space image (Montreal Neurological Institute, Montreal, QC, Canada) using FLIRT^24,27^ and FNIRT.^28^ Functional images were first registered to subjects’ high-resolution structural images with boundary-based registration and structural images were non-linearly registered to the standard space image. Functional images were then warped into standard space with a warp resolution of 10 mm.

### Neurovascular coupling

Neurovascular coupling was assessed in the occipital cortex using visual stimulation, as this region is a primary site of predilection for early CAA pathology.^29–31^ Unlike other regions such as the motor cortex, the occipital cortex shows early, robust impairment of neurovascular coupling in both sporadic and hereditary CAA.^5–7,32,33^ This approach maximizes the sensitivity for detecting early-stage NVC deficits.

Following a validated method,^34^ the preprocessed visually stimulated BOLD fMRI scans were analyzed to extract three neurovascular coupling measures. First, a subject-level general linear model (GLM) (FEAT, FSL) identified the 10% most visually activated voxels, which defined a subject-specific region of interest (ROI). Individual ROIs were chosen to optimize signal-to-noise ratio and account for individual anatomical variability, ensuring measurements are extracted from the most responsive tissue and providing greater precision than standardized group-level masks. This method has demonstrated high reproducibility and test-retest reliability in the CAA population.^34^ Next, average BOLD time series were extracted from the subject-specific ROI, segmented into the stimulus and rest blocks, and converted to percentage signal change using the mean value of all blocks as baseline. Finally, a trapezoidal function was fitted to the resulting time series using the TrapFit algorithm (https://github.com/jjhbw/TrapFit/) in R (The R foundation for Statistical Computing, Vienna, Austria, version 1.2.5033, https://www.rproject.org/). This process yielded three measures: The BOLD amplitude was the peak response from baseline, the time to peak (TTP) was the time from stimulus onset to the trapezoid ceiling, and the time to baseline (TTB) was the duration from the stimulus offset back to baseline. NVC measures were extracted for each of the three fMRI scans separately and then averaged.

### Functional connectivity

To examine brain function, two common approaches to study functional connectivity were used. First, functional connectivity within large-scale brain systems was investigated using a standard set of resting-state network templates. Second, a graph theoretical approach was applied where functional connectivity was defined on a finer spatial scale to examine regional effects and to quantify network properties of the whole brain functional network.

### Resting-state networks

A resting-state network (RSN) can be defined as a set of brain regions that show highly synchronized activity in a resting state (i.e., while the subject is not performing any task). These brain regions are said to be functionally connected.^8,9^ A number of RSNs have been consistently identified across study populations using independent component (ICA) analysis.^11,35,36^ A drawback of ICA is that each time the algorithm is run, slightly different components (RSNs) will be generated, hampering the generalizability of RSNs and comparability of findings between studies. To overcome this limitation, standardized network templates can be used to ensure reliable and consistent decomposition of the RSNs. We used validated standardized network templates provided by Smith et al.^11^ specifying ten resting-state networks in the brain: visual medial network, visual occipital network, visual lateral network, default mode network, cerebellar network, sensorimotor network, auditory network, executive control network, frontoparietal right network and frontoparietal left network. These networks have been associated with specific brain functions, such as sensory processing and cognitive function.^10,11^ Within-network functional connectivity was calculated using a dual regression approach in FSL. Preprocessed resting-state fMRI scans were first entered into a spatial regression to obtain subject-specific timeseries for twelve templates: 10 RSNs plus a cerebrospinal fluid (CSF) and white matter template for nuisance regression. Subject-specific timeseries were then entered into a temporal regression to obtain subject-specific spatial maps of each template. Resulting spatial maps contained a *z* score per voxel, indicating how well the voxel fitted the specific RSN timeseries. Mean *z* scores were calculated for each RSN by averaging the *z* scores of all voxels belonging to the RSN with higher scores indicating higher functional connectivity.

### Graph theoretical approach

Graph theory is a mathematical approach that defines a network as a graph consisting of nodes that represent the elements of a network (small brain regions) and edges that represent the interaction (here: functional connectivity) between them.^37,38^ We used the fine functional parcellation of Seitzman et al.^39^ that divides the brain into 300 nodes and calculated the functional connectivity between them. Spheres were drawn around MNI coordinates provided by Seitzman et al.^39^ where cortical spheres (*n* = 239) had a 5 mm radius and subcortical (*n* = 34) and cerebellar spheres (*n* = 27) had a 4 mm radius. Functional connectivity between nodes was calculated using GraphVar (version 2.03a)^40^ implemented in MATLAB (version 2020b). For this, mean BOLD timeseries of all 300 nodes were extracted from each participants’ preprocessed resting-state scan. Nuisance regression was performed prior to timeseries extraction regressing mean timeseries of CSF and WM out of the data. Next, Pearson's correlation coefficient was calculated between mean timeseries of each node with all other nodes and saved into a connectivity matrix. A connectivity matrix is a different representation of a graph with columns and rows representing the nodes and matrix values representing the edges, that is the functional connectivity as defined by Pearson's correlation coefficient. The diagonal of the matrix, the correlation of the node with itself, was set to zero. This resulted in a matrix of 300 × 300 that contained 44,850 unique connectivity values ((*N*(*N*-1)) / 2) for each subject. Matrices were Fisher's *r*-to-*z* transformed. The connectivity matrix was used in further statistical analyses to examine local functional connections.

Further, graph theory can be used to quantify aspects of the structure and dynamics of the brain network. We calculated two common graph measures that reflect properties of integration and segregation of the whole-brain network: weighted global efficiency (GE) and weighted clustering coefficient (CC), respectively. GE provides information about how efficiently information can flow through the brain.^38,41,42^ CC provides information about the presence of specialized information processing clusters and cliquishness of the network.^38,42,43^ Before GE and CC can be calculated from the connectivity matrix, a threshold must be applied to make the matrix sparser and remove spurious connections.^38^ As graph measures can be affected by the number of edges in a graph, a proportional thresholding approach was used to control the matrix density, ensuring the same number of edges for each participant.^44^ A proportional threshold of 30% was chosen to create a sparser network that limits spurious connections while preserving whole-brain network integrity. This choice was based on our previous research in a similar group of older adults, which showed that network measures remained stable across the 20% to 50% density range.^45^ Using a single pre-defined threshold provides a representative measure of the network organization while preventing the inflation of Type I errors when testing across a wide range of density levels. GE and CC were then calculated using the Brain Connectivity Toolbox^38^ implemented in GraphVar.

### Statistical analyses

Statistical analyses were performed for two groups: the total study sample (*n* = 93) and a specific subgroup consisting of participants meeting the Boston criteria v2.0 for possible or probable CAA (*n* = 46). While the study sample is considered at risk for cerebral small vessel disease, this subgroup was analyzed separately to specifically investigate associations in patients with MRI evidence of CAA pathology. This way we could determine whether the results were attributable to CAA-specific pathology or were influenced by other comorbid cerebrovascular pathologies that might be present in the broader sample. Before analysis, all variables were checked for normality and transformed using a natural log transformation if necessary.

Associations between the neurovascular coupling measures and functional connectivity measures were examined using GLMs. In all instances, TTP, TTB and BOLD amplitude served as the independent variables. Two GLM models were applied to each analysis. The first model included age and sex as covariates, while the second model served as a sensitivity analysis with diagnosis group (cognitively normal, SCI, MCI, or dementia) added as a control variable to determine whether the associations were independent of clinical diagnosis. These models were implemented across three different scales of connectivity data.

For the RSNs, GLMs were constructed in SPSS (version 29.0) with the mean *z* score of each of the ten RSNs as the dependent variable. Similarly, GLMs were constructed with one of the two whole brain graph measures (GE or CC) as the dependent variable. To investigate local connectivity, an edgewise analysis was performed in GraphVar,^40^ using GLMs with 10,000 permutations and the functional connectivity matrix as the dependent variable. Given the large number of statistical tests, multiple comparisons were controlled for using a false discovery rate (FDR) correction via the Benjamini-Hochberg procedure with a significance threshold of 0.05. This correction was applied independently for the ten RSNs, the two global graph measures, and the 44,850 unique edges in the edgewise analysis.

## Results

A total of 93 participants were included in the study, and their characteristics are presented in Table 1. On average, participants were 71 ± 9 years old, most were male, and well educated. The cognitive status of the sample varied with Mini-Mental State Examination (MMSE) scores ranging from 20 to 30. MRI markers of CAA showed that six participants presented with intracerebral hemorrhage and approximately half of the participants had at least one lobar microbleed. Half of the sample classified as possible (*n* = 20) or probable CAA (*n* = 26) according to the Boston criteria v2.0. Approximately 10% of the participants had one or more deep microbleeds and about 40% presented with lacunar infarcts. The neurovascular coupling measures showed considerable variability across the sample, as detailed in Table 1.

**Table 1.** Characteristics of the study population (n = 93).

Characteristic		
**Demographic and clinical**		
Age, years	71.3	(9.0)
Sex, male	53	(57.0%)
Education, Verhage score	6	[1–7]
MMSE score	28	[20–30]
Diagnosis		
None	39	(41.9%)
SCI	22	(23.7%)
MCI	21	(22.6%)
Dementia^a^	11	(11.8%)
**Cardiovascular risk factors**		
Hypertension	23	(24.7%)
Hyperlipidemia^b^	27	(29.0%)
Diabetes mellitus^b^	10	(10.8%)
Current smoking	13	(14.0%)
Cardiovascular disease	27	(29.0%)
**MRI markers of CAA**		
1 or more ICH^c^	6	(6.5%)
Lobar microbleeds^b^		
1	20	(21.5%)
2 or more	25	(28.0%)
DPVS-CSO^d^, high (score 3 or 4)	60	(64.5%)
DPVS-BG^b^, high (score 3 or 4)	40	(43%)
Cortical superficial sideroses^b^	1	(1.1%)
Possible CAA	20	(21.5%)
Probable CAA	26	(28.0%)
**Classic CSVD markers**		
Deep microbleeds^b^		
1	5	(5.4%)
2 or more	4	(4.3%)
Lacunar infarcts		
1	22	(23.7%)
2 or more	15	(16.2%)
WMH volume^e^, cm^3^	5.1	[0.6–61.5]
**Neurovascular coupling measures**		
Time to peak, s	5.85	(1.49)
Time to baseline, s	7.75	(1.98)
BOLD amplitude, % signal change	1.02	(0.27)

Data are presented as mean (*SD*), median [range], or number (percentage) where appropriate. BOLD: blood oxygen level-dependent; CAA: cerebral amyloid angiopathy; CSVD: cerebral small vessel disease; DPVS-BG: dilated perivascular spaces in the basal ganglia; DPVS-CSO: dilated perivascular spaces in the centrum semiovale; ICH: intracerebral hemorrhage; MCI: mild cognitive impairment; MMSE: Mini-Mental State Examination; SCI: subjective cognitive impairment; WMH: white matter hyperintensity.

^a^ Probable Alzheimer's disease or mixed type dementia.

^b^ Missing for 1 participant.

^c^ Missing for 2 participants.

^d^ Missing for 5 participants.

^e^ Normalized for intracranial volume.

The general linear models revealed no significant associations between the neurovascular coupling measures (TTP, TTB, and BOLD amplitude) and the large-scale functional connectivity metrics. Specifically, no significant relationships were found for the mean functional connectivity within the ten standard RSNs or the graph theoretical measures of global efficiency and clustering coefficient. These results remained consistent in a sensitivity analysis adjusting for clinical diagnosis. Within the total study sample, the assumption of homogeneity of regression slopes of the GLM was violated for the right frontoparietal network (*F*(9, 75) = 3.21, *p* = 0.002). This interaction between diagnostic group and NVC measures indicated that the relationship between NVC and functional connectivity differed across diagnostic groups. Consequently, the pooled sensitivity analysis (adjusting for clinical diagnosis) was omitted for this network and instead group-stratified models were performed. While these models suggested that the interaction was driven by a distinct slope direction in the MCI group, these results did not pass the statistical threshold. Aside from this specific interaction, results remained consistent across the total study sample and the CAA subgroup. The standardized regression coefficients, 95% confidence intervals and corresponding *p*-values of all the GLMs for the large-scale functional connectivity measures are provided in the Supplemental Material. Results for the total study sample, including the group-stratified results, are presented in Supplemental Table 1 (analyses adjusted for age and sex) and Supplemental Table 2 (sensitivity analysis), while results for the CAA subgroup are detailed in Supplemental Tables 3 and 4. Supplemental Figure 1 illustrates the lack of significant associations for the large-scale functional connectivity measures, showing scatterplots of the neurovascular coupling measures against global efficiency, clustering coefficient, and the default mode network. The default mode network is shown as a representative example of the RSNs as it is one of the most widely studied networks.

**Figure 1.**
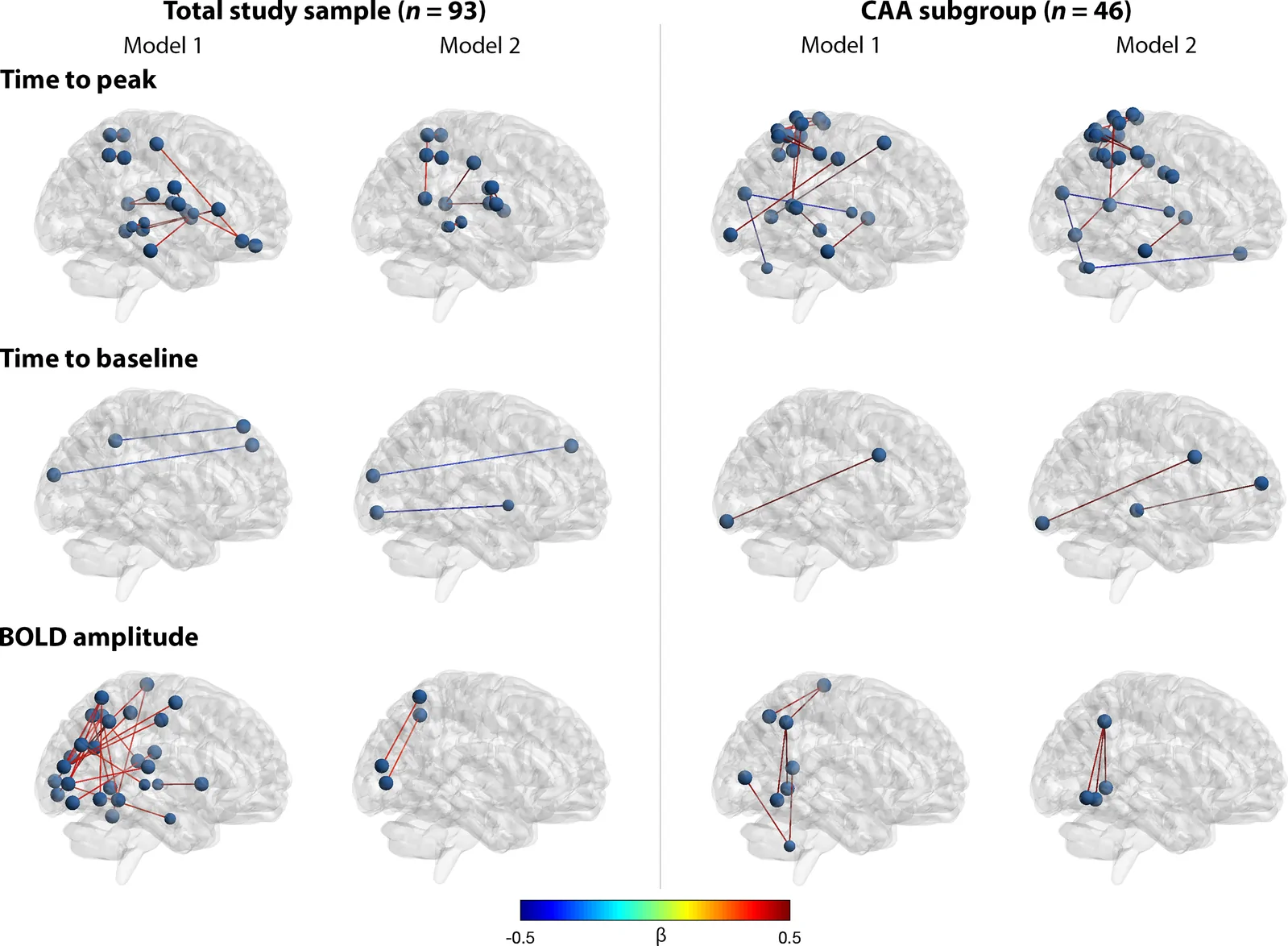
Significant local functional connections associated with neurovascular coupling measures. Significant edges (*p_FDR_* < 0.05) are displayed for time to peak (TTP), time to baseline (TTB) and blood oxygen level-dependent (BOLD) amplitude across both the total study sample (*n* = 93) and the CAA subgroup (*n* = 46). Results are shown for the primary model (adjusted for age and sex) and the secondary model (additionally adjusted for clinical diagnosis). Lines between nodes represent standardized regression weights, where positive and negative associations between the neurovascular coupling measure and functional connectivity strength are shown by different line colors (red and blue in the online version; corresponding gray shades in the print version). Brain networks were visualized with the BrainNet Viewer (http://www.nitrc.org/projects/bnv/).^46^

Edgewise analyses revealed a limited number of significant associations out of the 44,850 edges tested per NVC metric (all *p_FDR_* < 0.0001). Findings were largely consistent across the total study sample and the CAA subgroup. Figure 1 illustrates these significant edges for each NVC measure across both groups and both regression models.

Associations with TTB were the most sparse, with only two significant edges identified across these analyses. These involved frontoparietal and fronto-occipital connections, which exhibited a negative association with functional connectivity in the total sample, but a positive association within the CAA subgroup.

TTP was associated with a higher number of significant edges, primarily involving connections within and between parietal regions, as well as between parietal and subcortical areas. These edges generally showed a positive association with functional connectivity. In the total study sample, additional frontoparietal connections were observed, while the CAA subgroup exhibited negative associations involving occipital regions.

BOLD amplitude was significantly associated with parieto-occipital connectivity in both groups. In the total study sample, additional associations were found involving subcortical and frontal regions. However, these connections were no longer significant in the sensitivity model adjusting for clinical diagnosis. While the total study sample initially presented a higher number of significant edges than the CAA subgroup, this number decreased to similar levels in the sensitivity model. This suggests that the initial difference in edge significance may have been influenced by the clinical status of the participants.

## Discussion

In this study, we investigated the relationship between early CAA-related pathology and alterations in brain organization in a cohort of older adults. Our results demonstrate that early CAA-related pathology is associated with localized rather than global functional brain connectivity. While edgewise analyses revealed significant associations between neurovascular coupling measures (time to peak, time to baseline, and BOLD amplitude) and specific functional connections, no significant associations were identified for large-scale connectivity metrics. These global metrics included within-network connectivity across ten standard resting-state networks (RSNs) and whole brain graph theoretical measures of efficiency and segregation. Findings were consistent across the total study sample and the CAA subgroup and persisted after controlling for age, sex, and clinical diagnosis.

While prior research has identified functional connectivity disruptions in advanced CAA, the relationship between early CAA-related pathology and functional brain organization is less clear. Lower functional connectivity in the default mode network has been demonstrated in patients with probable sporadic CAA^19^ and across nearly all networks in symptomatic Dutch-type CAA.^18^ Our results suggest that such global alterations may not be present in earlier phases. The lack of significant findings at the global level in the current sample suggests that widespread functional connectivity disruption is a late-stage consequence of the disease. It is plausible that such global changes are preceded by a prolonged period of subtle, local network changes that are difficult to detect at a large-scale.

Indeed, our edgewise analysis revealed that early CAA-related pathology is associated with localized functional brain connectivity. Associations between specific functional connections and each NVC measure were identified that varied in their anatomical location and direction of the association. Time to peak was primarily associated with functional connectivity in parietal regions, whereas BOLD amplitude showed significant associations with parieto-occipital connectivity. Associations with time to baseline were more sparse, involving only two frontoparietal and fronto-occipital connections. These results correspond to the regional vulnerability patterns typically observed in CAA. The disease is characterized by a predilection for posterior brain regions, particularly the occipital cortex, and advancing anteriorly as disease severity increases.^29–31^ Furthermore, our results show that frontal brain areas are involved relatively early in the disease process. This is consistent with previous findings in presymptomatic Dutch-type CAA, where reduced functional connectivity was already evident in frontal regions.^18^ While our findings also implicate frontal connectivity at the localized level, this did not translate to the whole-network level as previously observed in the presymptomatic Dutch-type cohort. This discrepancy could stem from inherent differences between disease models. Dutch-type CAA typically exhibits a more aggressive clinical progression and earlier onset than sporadic CAA,^47–50^ which may result in more severe network-wide disruptions even in the early stages of the disease.

This study has several strengths, including the comprehensive assessment of both neurovascular coupling and functional connectivity using multiple robust methods in a well-characterized clinical cohort. The inclusion of a heterogeneous patient sample from a memory clinic represents both a strength and a limitation of this work. It is a strength in that it allows for the investigation of a broad continuum of pathological changes. However, this heterogeneity simultaneously serves as a limitation, as the presence of common co-morbid pathologies could potentially bias the results. Although similar results were found in the total study sample versus the CAA subgroup and findings remained consistent after controlling for clinical diagnosis, future research would benefit from targeting populations with higher diagnostic certainty to better distinguish CAA-specific effects from other comorbid conditions. For instance, studying hereditary CAA or autopsy-confirmed cases or incorporating molecular markers like amyloid-PET would allow for a more precise investigation. In addition, it is important to consider that the edgewise analyses required a very large number of statistical tests, which increases the risk of false positive findings. While we applied FDR correction to control for this risk, the number of significant edges found was small compared to the total of 44,850 connections tested. For this reason, these results should be interpreted with caution. Additionally, the relatively small sample size in some subgroups may have limited our statistical power. Nevertheless, the specific functional connections identified here could serve as a useful starting point for future studies. These findings now need to be corroborated in other independent study samples to validate the localized functional connections in relation to early CAA. Furthermore, the study's cross-sectional design limits our ability to make causal inferences. Future longitudinal studies following patients until a more definitive diagnosis is reached would provide deeper insight into early-stage disease mechanisms. Finally, our graph theoretical analysis relied on a single 30% network density threshold. Because graph theoretical measures are sensitive to the number of edges in a graph, a fixed density could obscure true network topology.^44^ To validate our findings, future studies could consider evaluating a range of thresholds to assess stability of the observed associations.

An intrinsic limitation of this study concerns the BOLD signal from which both the neurovascular coupling and functional connectivity measures were derived. Although functional connectivity is often interpreted as a measure of neuronal activity, the BOLD signal is a complex signal containing both neural and vascular components. This dual nature of the BOLD signal is a critical factor when interpreting functional connectivity in patients with vascular pathology, which may challenge the interpretability of the findings. Theoretically, vascular impairment may alter the temporal correlations between brain regions, potentially leading to lower functional connectivity estimates^51,52^ and thereby masking true neural effects or mimicking network associations. Yet, if impaired neurovascular coupling was biasing the functional connectivity measurements, we would expect to find strong associations between the two metrics across scales, particularly within the mainly occipitally located visual networks. The absence of such an association suggests that neurovascular coupling and functional connectivity capture different physiological processes of the disease, rather than being redundant measures of the same underlying vascular impairment. Nonetheless, the relationship between stimulation-induced and resting-state BOLD signals is not yet clear^51^ and remains an area for further investigation. Future research incorporating arterial spin labelling could help to further disentangle these neural and vascular components.

The results of this study suggest that impaired neurovascular coupling and altered functional connectivity may reflect distinct or intricately related pathophysiological disease processes. Future research should prioritize longitudinal studies to track the progression of neurovascular coupling and functional connectivity over time across different stages of CAA to determine if early vascular deficits predict later changes in functional brain organization. This may be especially informative in “pure” CAA populations to minimize confounding from other neurodegenerative diseases, such as Alzheimer's disease. A clearer understanding of the specific impact of CAA pathology will inform not only our knowledge of CAA itself, but also our understanding of comorbid Alzheimer's disease. Finally, while the occipital cortex represents a critical area due to the posterior-dominant predilection of CAA pathology,^29–31^ research utilizing neurovascular coupling measures across the entire brain and tests of vascular function independent of neural activity, such as through techniques like CO_2_ inhalation, could provide a more comprehensive understanding of the local interplay between vascular health and functional networks.

In conclusion, our findings suggest that functional connectivity alterations associated with early CAA-related pathology originate at a localized scale. Significant global alterations in functional connectivity likely only emerge as a consequence of more advanced, later-stage pathological processes. Therefore, our findings highlight the need for comprehensive, longitudinal, multi-scale studies that track the progression of CAA pathology, the transition from localized deficits to global network failure and clinical outcomes. This is essential to establish causality and understand the temporal sequence of vascular and functional decline in CAA.

## Supplemental Material

sj-docx-1-alz-10.1177_13872877261469189 - Supplemental material for Early cerebral amyloid angiopathy-related pathology is associated with localized functional brain connectivity Supplemental material, sj-docx-1-alz-10.1177_13872877261469189 for Early cerebral amyloid angiopathy-related pathology is associated with localized functional brain connectivity by Nadieh Drenth, Suzanne E. van Dijk, Anne Hafkemeijer, Serge A.R.B. Rombouts, Jeroen van der Grond and Sanneke van Rooden in Journal of Alzheimer's Disease
