# Supplementary material for: Early cerebral amyloid angiopathy-related pathology is associated with localized functional brain connectivity
Source: J Alzheimers Dis. 2026 Jul 23;113(1):414–23. doi: 10.1177/13872877261469189 (PMC13499917; doi:10.1177/13872877261469189)
Supplement: Supplementary file 1 [file sj-docx-1-alz-10.1177_13872877261469189.docx]

**Supplemental Material**

**Early cerebral amyloid angiopathy-related pathology is associated with localized functional brain connectivity**

# Supplemental Results

**Supplemental Table 1.** Associations between neurovascular coupling measures and functional connectivity adjusted for age and sex in the whole study sample (n=93).

|  | Time to peak | | Time to baseline | | BOLD amplitude | |
| --- | --- | --- | --- | --- | --- | --- |
|  | β [95% CI] | *p* | β [95% CI] | *p* | β [95% CI] | *p* |
| **RSNs** |  |  |  |  |  |  |
| Visual medial | 0.14 [-0.07 to 0.35] | 0.190 | -0.04 [-0.25 to 0.17] | 0.692 | -0.02 [-0.24 to 0.21] | 0.882 |
| Visual occipital | 0.00 [-0.21 to 0.22] | 0.973 | 0.00 [-0.21 to 0.21] | 0.995 | 0.11 [-0.12 to 0.34] | 0.333 |
| Visual lateral | 0.02 [-0.19 to 0.23] | 0.848 | 0.02 [-0.19 to 0.23] | 0.829 | 0.16 [-0.07 to 0.38] | 0.169 |
| Default mode | 0.08 [-0.12 to 0.29] | 0.423 | 0.04 [-0.16 to 0.24] | 0.722 | 0.03 [-0.19 to 0.24] | 0.821 |
| Cerebellar | 0.17 [-0.05 to 0.38] | 0.120 | -0.07 [-0.28 to 0.14] | 0.525 | -0.01 [-0.24 to 0.22] | 0.922 |
| Sensorimotor | 0.11 [-0.11 to 0.32] | 0.325 | -0.03 [-0.23 to 0.18] | 0.782 | -0.02 [-0.24 to 0.21] | 0.894 |
| Auditory | 0.23 [0.03 to 0.44] | 0.026 | -0.14 [-0.34 to 0.06] | 0.169 | 0.10 [-0.12 to 0.31] | 0.367 |
| Executive control | 0.17 [-0.04 to 0.38] | 0.120 | 0.00 [-0.20 to 0.21] | 0.977 | 0.05 [-0.18 to 0.27] | 0.685 |
| Frontoparietal right^a^ |  |  |  |  |  |  |
| Control subjects | -0.10 [-0.55 to 0.31] | 0.576 | 0.05 [-0.30 to 0.42] | 0.738 | 0.36 [0.02 to 0.74] | 0.039 |
| SCI | -0.42 [-1.01 to 0.10] | 0.102 | 0.15 [ -0.54 to 0.97] | 0.557 | 0.05 [-0.65 to 0.77] | 0.864 |
| MCI | 0.49 [0.10 to 0.70] | 0.012 | -0.45 [-0.66 to -0.07] | 0.019 | -0.59 [-1.04 to -0.27] | 0.002 |
| Dementia | 0.46 [-0.42 to 1.19] | 0.276 | -0.13 [-0.75 to 0.51] | 0.647 | 0.62 [ -0.29 to 1.77] | 0.124 |
| Frontoparietal left | 0.12 [-0.1 to 0.33] | 0.282 | -0.03 [-0.24 to 0.18] | 0.802 | -0.07 [-0.29 to 0.16] | 0.568 |
| **Graph theoretical measures** |  |  |  |  |  |  |
| Global efficiency | 0.16 [-0.05 to 0.37] | 0.124 | -0.04 [-0.24 to 0.16] | 0.689 | 0.19 [-0.03 to 0.41] | 0.092 |
| Clustering coefficient | 0.11 [-0.10 to 0.32] | 0.287 | -0.03 [-0.23 to 0.18] | 0.779 | 0.22 [0.00 to 0.44] | 0.046 |

Reported *p*-values are uncorrected. None of the associations were significant (*p_FDR_* < 0.05). BOLD: blood oxygen level-dependent; CI: confidence interval; RSN: resting-state network; SCI: subjective cognitive impairment; MCI: mild cognitive impairment

^a^ For the right frontoparietal network, the assumption of homogeneity of regression slopes was violated (*F*(9, 75) = 3.21, *p* = 0.002), necessitating separate analysis for each clinical group.

**Supplemental Table 2.** Associations between neurovascular coupling measures and functional connectivity adjusted for age, sex and diagnosis group in the whole study sample (n=93).

|  | Time to peak | | Time to baseline | | BOLD amplitude | |
| --- | --- | --- | --- | --- | --- | --- |
|  | β [95% CI] | *p* | β [95% CI] | *p* | β [95% CI] | *p* |
| **RSNs** |  |  |  |  |  |  |
| Visual medial | 0.15 [-0.07 to 0.36] | 0.171 | -0.06 [-0.27 to 0.15] | 0.579 | -0.05 [-0.28 to 0.19] | 0.703 |
| Visual occipital | 0.00 [-0.22 to 0.22] | 0.996 | 0.01 [-0.21 to 0.23] | 0.927 | 0.13 [-0.12 to 0.37] | 0.297 |
| Visual lateral | 0.02 [-0.19 to 0.24] | 0.834 | 0.02 [-0.20 to 0.23] | 0.875 | 0.15 [-0.09 to 0.39] | 0.224 |
| Default mode | 0.09 [-0.11 to 0.30] | 0.375 | 0.01 [-0.19 to 0.22] | 0.910 | -0.01 [-0.24 to 0.22] | 0.910 |
| Cerebellar | 0.18 [-0.04 to 0.39] | 0.102 | -0.09 [-0.31 to 0.12] | 0.392 | -0.05 [-0.29 to 0.19] | 0.669 |
| Sensorimotor | 0.11 [-0.10 to 0.32] | 0.301 | -0.04 [-0.26 to 0.17] | 0.682 | -0.04 [-0.28 to 0.20] | 0.748 |
| Auditory | 0.25 [0.05 to 0.45] | 0.014 | -0.19 [-0.39 to 0.01] | 0.061 | 0.02 [-0.21 to 0.24] | 0.891 |
| Executive control | 0.18 [-0.03 to 0.39] | 0.100 | 0.03 [-0.24 to 0.19] | 0.819 | 0.00 [-0.24 to 0.24] | 0.981 |
| Frontoparietal right^a^ | - | - | - | - | - | - |
| Frontoparietal left | 0.11 [-0.10 to 0.33] | 0.305 | -0.02 [-0.23 to 0.20] | 0.894 | -0.05 [-0.29 to 0.19] | 0.703 |
| **Graph theoretical measures** |  |  |  |  |  |  |
| Global efficiency | 0.18 [-0.03 to 0.38] | 0.087 | -0.09 [-0.29 to 0.12] | 0.412 | 0.12 [-0.11 to 0.35] | 0.312 |
| Clustering coefficient | 0.13 [-0.08 to 0.33] | 0.234 | -0.06 [-0.27 to 0.15] | 0.546 | 0.17 [-0.06 to 0.40] | 0.153 |

Reported *p*-values are uncorrected. None of the associations were significant (*p_FDR_* < 0.05). BOLD: blood oxygen level-dependent; CI: confidence interval, RSN = resting-state network.

^a^ For the right frontoparietal network, the overall model with group as a covariate was omitted due to the violation of the homogeneity of regression slopes assumption. Instead, group-specific associations are presented in Supplemental Table 1.

**Supplemental Table 3.** Associations between neurovascular coupling measures and functional connectivity adjusted for age and sex in the CAA subgroup (n=46).

|  | Time to peak | | Time to baseline | | BOLD amplitude | |
| --- | --- | --- | --- | --- | --- | --- |
|  | β [95% CI] | *p* | β [95% CI] | *p* | β [95% CI] | *p* |
| **RSNs** |  |  |  |  |  |  |
| Visual medial | 0.27 [-0.05 to 0.58] | 0.092 | -0.16 [-0.47 to 0.15] | 0.303 | -0.13 [-0.44 to 0.19] | 0.423 |
| Visual occipital | 0.11 [-0.23 to 0.44] | 0.525 | -0.03 [-0.35 to 0.30] | 0.876 | 0.05 [-0.28 to 0.38] | 0.777 |
| Visual lateral | 0.00 [-0.32 to 0.32] | 0.999 | 0.20 [-0.11 to 0.52] | 0.202 | 0.16 [-0.16 to 0.48] | 0.319 |
| Default mode | 0.26 [-0.06 to 0.57] | 0.108 | -0.02 [-0.32 to 0.29] | 0.920 | -0.01 [-0.33 to 0.30] | 0.931 |
| Cerebellar | 0.19 [-0.13 to 0.52] | 0.243 | 0.00 [-0.32 to 0.32] | 0.990 | 0.07 [-0.26 to 0.39] | 0.673 |
| Sensorimotor | 0.38 [0.08 to 0.68] | 0.015 | -0.17 [-0.46 to 0.12] | 0.252 | -0.18 [-0.48 to 0.12] | 0.224 |
| Auditory | 0.20 [-0.11 to 0.51] | 0.207 | -0.05 [-0.36 to 0.25] | 0.721 | 0.00 [-0.31 to 0.30] | 0.976 |
| Executive control | 0.30 [-0.02 to 0.63] | 0.067 | -0.16 [-0.48 to 0.16] | 0.322 | -0.02 [-0.35 to 0.30] | 0.890 |
| Frontoparietal right | 0.15 [-0.18 to 0.48] | 0.354 | -0.03 [-0.35 to 0.29] | 0.850 | 0.26 [-0.07 to 0.59] | 0.118 |
| Frontoparietal left | 0.19 [-0.14 to 0.52] | 0.246 | 0.12 [-0.20 to 0.44] | 0.457 | -0.10 [-0.43 to 0.23] | 0.530 |
| **Graph theoretical measures** |  |  |  |  |  |  |
| Global efficiency | 0.20 [-0.13 to 0.52] | 0.236 | 0.03 [-0.29 to 0.35] | 0.843 | -0.03 [-0.35 to 0.30] | 0.876 |
| Clustering coefficient | 0.13 [-0.20 to 0.47] | 0.434 | 0.05 [-0.28 to 0.37] | 0.782 | 0.05 [-0.29 to 0.38] | 0.786 |

Reported *p*-values are uncorrected. None of the associations were significant (*p_FDR_* < 0.05). BOLD: blood oxygen level-dependent; CI: confidence interval; RSN: resting-state network

**Supplemental Table 4.** Associations between neurovascular coupling measures and functional connectivity adjusted for age, sex and diagnosis group in the CAA subgroup (n=46).

|  | Time to peak | | Time to baseline | | BOLD amplitude | |
| --- | --- | --- | --- | --- | --- | --- |
|  | β [95% CI] | *p* | β [95% CI] | *p* | β [95% CI] | *p* |
| **RSNs** |  |  |  |  |  |  |
| Visual medial | 0.28 [-0.05 to 0.60] | 0.092 | -0.16 [-0.48 to 0.15] | 0.297 | -0.15 [-0.51 to 0.21] | 0.406 |
| Visual occipital | 0.12 [-0.22 to 0.46] | 0.475 | -0.04 [-0.36 to 0.29] | 0.823 | -0.02 [-0.39 to 0.36] | 0.934 |
| Visual lateral | 0.01 [-0.32 to 0.34] | 0.934 | 0.19 [-0.13 to 0.51] | 0.232 | 0.10 [-0.27 to 0.47] | 0.573 |
| Default mode | 0.29 [-0.02 to 0.60] | 0.070 | -0.04 [-0.34 to 0.26] | 0.792 | -0.15 [-0.50 to 0.20] | 0.401 |
| Cerebellar | 0.20 [-0.14 to 0.53] | 0.243 | 0.00 [-0.33 to 0.32] | 0.994 | 0.05 [-0.32 to 0.43] | 0.784 |
| Sensorimotor | 0.38 [0.08 to 0.69] | 0.015 | -0.18 [-0.47 to 0.12] | 0.240 | -0.22 [-0.56 to 0.12] | 0.202 |
| Auditory | 0.23 [-0.08 to 0.54] | 0.143 | -0.08 [-0.38 to 0.22] | 0.604 | -0.13 [-0.48 to 0.21] | 0.442 |
| Executive control | 0.33 [0.01 to 0.66] | 0.045 | -0.18 [-0.50 to 0.14] | 0.254 | -0.15 [-0.51 to 0.22] | 0.417 |
| Frontoparietal right | 0.17 [-0.16 to 0.50] | 0.310 | -0.04 [-0.37 to 0.28] | 0.789 | 0.19 [-0.19 to 0.56] | 0.317 |
| Frontoparietal left | 0.19 [-0.15 to 0.53] | 0.265 | 0.12 [-0.21 to 0.45] | 0.454 | -0.09 [-0.47 to 0.29] | 0.645 |
| **Graph theoretical measures** |  |  |  |  |  |  |
| Global efficiency | 0.23 [-0.10 to 0.55] | 0.167 | 0.01 [-0.31 to 0.33] | 0.963 | -0.16 [-0.53 to 0.21] | 0.386 |
| Clustering coefficient | 0.16 [-0.18 to 0.49] | 0.357 | 0.03 [-0.30 to 0.36] | 0.872 | -0.06 [-0.44 to 0.32] | 0.758 |

Reported *p*-values are uncorrected. None of the associations were significant (*p_FDR_* < 0.05). BOLD: blood oxygen level-dependent; CI: confidence interval; RSN: resting-state network

**Supplemental Figure 1.** Scatterplots of neurovascular coupling measures time to peak, time to baseline and BOLD amplitude against mean functional connectivity within the default mode network, global efficiency, and clustering coefficient in the total study sample. Standardized regression coefficients with 95% confidence intervals are presented for each panel. None of the associations were significant. FC: functional connectivity; DMN: default mode network; BOLD: blood oxygen level-dependent.
